# Lysosome-Dependent LXR and PPARδ Activation Upon Efferocytosis in Human Macrophages

**DOI:** 10.3389/fimmu.2021.637778

**Published:** 2021-05-07

**Authors:** Ana Carolina Mota, Monica Dominguez, Andreas Weigert, Ryan G. Snodgrass, Dmitry Namgaladze, Bernhard Brüne

**Affiliations:** ^1^ Institute of Biochemistry I, Faculty of Medicine, Goethe-University Frankfurt, Frankfurt, Germany; ^2^ Fraunhofer Institute for Translational Medicine and Pharmacology ITMP, Frankfurt, Germany; ^3^ German Cancer Consortium (DKTK), Partner Site Frankfurt, Frankfurt, Germany; ^4^ Frankfurt Cancer Institute, Goethe-University Frankfurt, Frankfurt, Germany

**Keywords:** macrophages, efferocytosis, apoptosis, peroxisome proliferator-activated receptor, liver X receptor

## Abstract

Efferocytosis is critical for tissue homeostasis, as its deregulation is associated with several autoimmune pathologies. While engulfing apoptotic cells, phagocytes activate transcription factors, such as peroxisome proliferator-activated receptors (PPAR) or liver X receptors (LXR) that orchestrate metabolic, phagocytic, and inflammatory responses towards the ingested material. Coordination of these transcription factors in efferocytotic human macrophages is not fully understood. In this study, we evaluated the transcriptional profile of macrophages following the uptake of apoptotic Jurkat T cells using RNA-seq analysis. Results indicated upregulation of PPAR and LXR pathways but downregulation of sterol regulatory element-binding proteins (SREBP) target genes. Pharmacological inhibition and RNA interference pointed to LXR and PPARδ as relevant transcriptional regulators, while PPARγ did not substantially contribute to gene regulation. Mechanistically, lysosomal digestion and lysosomal acid lipase (LIPA) were required for PPAR and LXR activation, while PPARδ activation also demanded an active lysosomal phospholipase A_2_ (PLA2G15). Pharmacological interference with LXR signaling attenuated ABCA1-dependent cholesterol efflux from efferocytotic macrophages, but suppression of inflammatory responses following efferocytosis occurred independently of LXR and PPARδ. These data provide mechanistic details on LXR and PPARδ activation in efferocytotic human macrophages.

## Introduction

Macrophage (Mφ) engulfment of apoptotic cells (AC), a process known as efferocytosis, promotes resolution of inflammation and tissue repair, while restricting autoreactive immune responses (1). Efferocytosis is a coordinated sequence of events, which starts with recognition of AC and culminates in their phagocytosis, followed by phagolysosomal processing (2). Effective clearance of AC is essential for maintaining tissue homeostasis. More recent studies pointed to its importance in resolution of inflammation, immune tolerance, and cancer development (3). Therefore, a better understanding of efferocytosis is key to comprehend major pathophysiological processes.

Phagolysosomal processing of AC is central to handle ingested material (4, 5). It creates an overload with macromolecular species that Mφ either use or efflux (2). For example, efferocytotic accumulation of lipids from engulfed cells generates ligands for nuclear receptors, which regulate lipid metabolism in Mφ, including liver X receptors (LXRα and LXRβ) and peroxisome proliferator-activated receptors (PPARα, δ or γ) (6). LXRs, PPARγ and PPARδ are well-characterized transcriptional regulators that coordinate the clearance of AC as well as anti-inflammatory responses of Mφ (7–9).

LXRs are activated by oxysterols or cholesterol biosynthetic intermediates that sense accumulated cellular cholesterol (7, 8, 10). LXR activation induces cholesterol efflux by increasing the expression of ATP-binding cassette (ABC) transporters (ABCA1 and ABCG1) (11). PPARs dominate the transcriptional control of fatty acid metabolism (6, 8). Moreover, LXRs, PPARδ and PPARγ also regulate Mφ inflammatory responses (2, 8, 12, 13). Even though PPARs and LXRs have well-established functions during efferocytosis, their activation upon lysosomal processing of apoptotic material is not fully understood. Recently, lysosomal acid lipase (LIPA) was suggested to be necessary for LXR activation in efferocytes (14), while mechanisms generating PPAR ligands upon AC engulfment remain unclear.

Most of the previous work concerning the roles of LXRs and PPARs in efferocytosis employed animal models, often using mice with a Mφ-specific deficiency of individual transcription factors (7, 8). LXRα/β- and PPARδ-deficient Mφ reduce expression of the efferocytotic receptor Mer to about 25 to 40%, which largely attenuates the uptake of AC (7, 8). Thus, gene expression changes due to a constitutive deficiency of nuclear receptors substantially alter efferocytotic capabilities of Mφ. Therefore, model systems using a gene knockout strategy are only of limited predictive value when addressing the relevance of acute activation of LXRs and PPARs during efferocytosis. Along these lines, mechanistic work on these transcription factors using primary human Mφ is sparsely described. Considering substantial differences between murine and human Mφ, and the need for translational studies makes human Mφ a relevant test system.

Our study aimed at describing activation of PPARs and LXRs in efferocytotic primary human Mφ and explores how their activation shapes metabolism and inflammatory responses. The transcriptional LXR and PPAR responses in efferocytotic Mφ demand lysosomal processing of ingested material and activities of LIPA and the lysosomal phospholipase A_2_.

## Materials and Methods

### Cell Culture and Reagents

Jurkat T cells were purchased from ATCC and maintained in RPMI 1640 (Gibco) supplemented with 10% heat-inactivated FCS, 100 U/mL penicillin and 100 μg/mL streptomycin. Human monocytes were isolated from commercially available buffy coats from anonymous donors (DRK-Blutspendedienst Baden-Württemberg - Hessen, Institut für Transfusionsmedizin und Immunhämatologie, Frankfurt, Germany) using Ficoll (Biochrom) density centrifugation. Monocytes were isolated from peripheral blood mononuclear cells (PBMCs) using positive selection with CD14 antibody-coupled magnetic beads (MACS Miltenyi Biotec) and LS columns (MACS Miltenyi Biotec) following the manufacturer’s protocol.

CD14^+^ monocytes were differentiated in macrophage serum-free medium (ThermoFisher Scientific) supplemented with 100 U/mL penicillin, 100 μg/mL streptomycin and 50 ng/mL of macrophage colony-stimulating factor (M-CSF) (Immunotools) for 7 days followed by culture in RPMI 1640 medium supplemented with 10% heat-inactivated FCS, 100 U/mL penicillin and 100 μg/mL streptomycin. When indicated, cells were treated with 1 µM of T0070907 (Cayman Chemical), 100 nM GW501516 (Cayman Chemical), 1 µM of Rosiglitazone (Sigma-Aldrich), 10 nM of T0901317 (Tocris Bioscience), 1 µM of GSK3787 (Cayman Chemical), 5 µM of GSK2033 (Sigma-Aldrich), 100 nM of concanamycin (Santa Cruz Biotechnology), 10 µM of isopropyl dodecylphosphonofluoridate (IDFP) (Cayman Chemical), 10 µM of probucol (Cayman Chemical) and 10 ng/mL of LPS (Sigma-Aldrich). Treatments did not compromise Mφ viability.

### *In Vitro* Efferocytosis Assay

To induce apoptosis, Jurkat cells were seeded in 10 cm dishes in serum-free RPMI 1640 medium and exposed to 100 mJ/cm^2^ UV-C (254nm) irradiation (UVP Crosslinker CL-1000, Jena Analytik) followed by incubation for 3 hours at 37°C with 5% CO_2_. Human Mφ were stimulated with apoptotic Jurkat cells at a 1:3 ratio in 6-well plates for 3 or 6 hours. Upon efferocytosis, non-phagocytosed cells were removed and Mφ were washed 3x with PBS, followed by incubation with RPMI 1640 supplemented with 10% heat-inactivated FCS, 100 U/mL penicillin and 100 μg/mL streptomycin for 3, 6, 9 and 21 hours.

### Apoptosis Analysis

Apoptosis of Jurkat cells was assessed with Annexin V-FITC/propidium Iodide (PI) double staining. Upon UV treatment, Jurkat cells were washed with PBS and resuspended in Annexin V Binding Buffer (10 mM HEPES, pH 7.4, 150 mM NaCl, 2.5 mM CaCl_2_ in PBS) with 1 µg/mL of Annexin V-FITC (Immunotools) and 1 µg/mL of PI (Thermofisher Scientific). Samples were incubated at room temperature in the dark for 15 minutes followed by flow cytometry analysis with a LSRII/Fortessa flow cytometer (BD Biosciences).

### Efferocytosis Analyses

Jurkat cells were labelled using CellTracker™ Orange CMRA (ThermoFisher Scientific) according to the manufacturer’s recommendations. Mφ were seeded onto 8-well chambered coverslips (µ-slide, ibidi GmbH), stained with CFSE Cell Division Tracker Kit (Biolegend) and incubated with labelled apoptotic Jurkat cells at a 1:3 ratio. Upon removal of the non-phagocytosed cells, Mφ were analyzed by fluorescence imaging using a Plan-Apochromat 20x long range objective on a Zeiss LSM800 confocal microscope driven by the Zen 2009 software (Carl Zeiss) or by flow cytometry with a LSRII/Fortessa flow cytometer (BD Biosciences).

### NGS Library Preparation and RNA Sequencing

Total RNA of non-treated and efferocytotic Mφ (6 biological replicates each) was isolated using RNeasy Micro Kit (Qiagen) according to the manufacturer’s protocol. cDNA library preparation was carried out using QuantSeq 3’ mRNA-Seq Library Prep Kit FWD from Illumina (Lexogen) according to the manufacturer’s procedure. RNA and DNA quantification was done using Qubit cDNA HS Assay Kits (ThermoFisher Scientific) and quality control was performed using an Agilent 2100 Bioanalyzer with RNA Nano Chip (Agilent) as well as High Sensivity DNA chips (Agilent). Libraries were diluted and denatured according to the Illumina Denature and Dilute Libraries Guide, followed by mixing with 1% Phix Control (Illumina). 12 Libraries were loaded on one sequencing cartridge of the TG NextSeq 500/550 High Output Kit v2 (75 cycles) (Illumina) and RNA sequencing was performed on a NextSeq500 system (Illumina).

### RNA-seq Data Processing, Differential Expression Analysis and GSEA Analysis

Statistics of the individual RNA sequence data sets were monitored by FastQC analysis. RNA-seq data processing and differential expression analysis was performed using the QuantSeq data analysis pipeline from Bluebee Genomics analysis platform following manufacturer’s instructions. Genes significantly regulated by efferocytosis were extracted by setting a threshold based on the average number of reads to avoid Jurkat T cell-specific genes (set at the level of CD3). 39.555 genes with lower number of reads were removed. After this selection, a 20.646 gene set was further analyzed using Gene Set Enrichment Analysis 3.0. Gene list of up- and down-regulated genes of efferocytotic versus non-treated MΦ was generated using the following inclusion criteria: |log_2_FC|>1 and Padjusted≤0.05, and normalized base mean above 50. The lists were ranked based on adjusted *P* value. The RNA-seq data are available at the Gene Expression Omnibus database under accession number GSE169160.

### RNA Extraction and Q-PCR

Total RNA was isolated with peqGOLD RNAPure reagent (PeqLab Biotechnology) according to manufacturer’s recommendations followed by reverse transcription using Maxima first-strand cDNA synthesis kit (ThermoFisher Scientific). Quantitative real-time PCR (Q-PCR) assays were performed with PowerUp SYBR Green Master Mix (Applied Biosystems) using Quant Studio Real Time PCR System (Applied Biosystems). Relative transcript amounts were quantified using the Δct method with β-microglobulin (βMG) as a housekeeping gene and normalized to the untreated or apoptotic cell-treated controls.

### siRNA Transfection

Knockdowns of PPARδ, PPARγ, LIPA and PLA2G15 were performed using transfections with 50 nM siRNA (siGENOME human SMARTpool, Thermo Scientific) and Hyperfect transfection reagent (Qiagen) according to manufacturer’s recommendations. Cells were treated 96 hours post-transfection.

### Western Blot Analysis

Cell pellets were harvested in lysis buffer (50 mM Tris-HCl, pH 8, 150 mM NaCl, 5 mM EDTA, 10 mM NaF, 1 mM Na_3_VO_4_, 0.5% NP-40, 1 mM PMSF, protease inhibitor cocktail). Protein lysates were sonicated and centrifuged at 12.000g for 10 minutes at 4°C. Supernatants were heat-denatured at 95°C, separated on SDS-PAGE gels, and transferred onto nitrocellulose membranes. For ABCA1 and ABCG1 Western blots, the heating step was omitted. Primary antibodies directed against ABCA1 (#NB400105SS, Novus Biologicals), ABCG1 (#13578-1-AP, Proteintech), CPT1A (#15184-1-AP, Proteintech) and nucleolin (#sc-55486, Santa Cruz Biotechnology) were used followed by IRDye 680 or IRDye 800-coupled secondary antibodies (LICOR Biosciences). Blots were visualized using LICOR ODYSSEY scanner, and analyzed by Image Studio™ Lite software (LICOR Biosciences).

### Seahorse Measurements

Cellular oxygen consumption rates (OCR) and extracellular acidification rates (ECAR) were analyzed using a Seahorse 96 extracellular flux analyzer (Agilent). Human Mφ were plated in Seahorse 96-well cell culture plates one day before measurements and equilibrated in Krebs-Henseleit buffer (111 mM NaCl, 4.7 mM KCl, 1.25 mM CaCl_2_, 2 mM MgSO_4_, 1.2 mM Na_2_HPO_4_) supplemented with 11 mM L-glucose and 0.2 mM L-glutamine for 1 hour prior to the assay. Cells were treated with 2.5 µM oligomycin (Sigma-Aldrich), 1 µM carbonyl cyanide m-chlorophenyl hydrazine (CCCP, Sigma-Aldrich), 1 µM rotenone (Sigma-Aldrich), and 1 µg/mL antimycin (Sigma-Aldrich).

### Cholesterol Efflux Measurements

Human Mφ were stimulated with apoptotic Jurkat cells at a 1:3 ratio in 6-well plates for 3 hours. Upon efferocytosis, non-phagocytosed cells were removed and Mφ were washed 3 times with PBS, followed by incubation with phenol red-free RPMI 1640 supplemented with 10 µg/mL of human recombinant ApoAI (Calbiochem), 100 U/mL penicillin and 100 μg/mL streptomycin for 21 hours. Cholesterol levels in the supernatant of efferocytotic Mφ were measured using the Amplex Red Cholesterol Assay Kit (Invitrogen) following the manufacturer’s protocol.

### Statistical Analysis

Statistical analysis was performed using GraphPad Prism 8.2. Data were analyzed using Student unpaired, two-tailed t test or by one-way ANOVA with Bonferoni multiple comparisons. Comparisons to normalized controls were analyzed using one-sample t test. Graphical data are presented as means ± SEM for at least three independent experiments. Asterisks indicate significant differences between experimental groups (*p<0.05, **p<0.01, ***p<0.005).

## Results

### LXR and PPAR Target Gene Induction Following Efferocytosis

**Figure 1:**
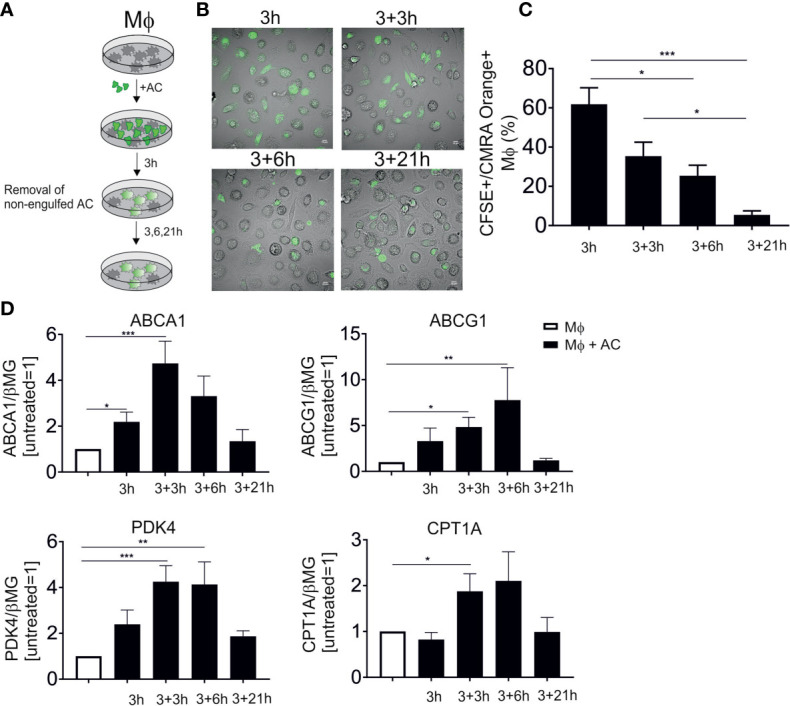
LXR and PPAR target gene induction following efferocytosis. **(A)** Scheme of our *in vitro* efferocytosis model. Mφ were exposed to apoptotic Jurkat cells (AC) for 3 hours, followed by removal of non-phagocytosed cells and subsequent incubation for 3, 6 and 21 hours. **(B)** Representative images of Mφ 3, 6, 9 and 24 hours post-efferocytosis of CMRA Orange-stained Jurkat cells (green). **(C)** Quantification of CFSE^+^/CMRA Orange^+^ Mφ upon efferocytosis of labelled Jurkat cells. **(D)** mRNA expression of LXR (ABCA1 and ABCG1) and PPAR (PDK4 and CPT1A) target genes at different time points post-efferocytosis. *P ≤ 0.05; **P ≤ 0.01; ***P ≤ 0.001.

PPARs and LXRs are well-established master transcriptional regulators of efferocytotic Mφ, although their functional input to shape the phenotype of human Mφ is only poorly understood. To follow LXR/PPAR activation, we set up an *in vitro* human efferocytosis model, consisting of monocyte-derived human Mφ and apoptotic Jurkat T cells (**Figure 1A**). Apoptosis of Jurkat cells was induced by UV-C exposure, which caused 75-85% apoptotic cells after 3 hours. To confirm time-dependent efferocytosis, CMRA Orange-labelled AC were added to Mφ for 3 hours, followed by the removal of the non-phagocytosed cells, and subsequent incubations in fresh medium for up to 24 hours. Confocal microscopy showed efficient efferocytosis after 3 and 6 hours when Mφ engulfed substantial amounts of apoptotic material (**Figure 1B**). Quantification of these results by flow cytometry revealed roughly 60% CFSE^+^/CMRA Orange^+^ Mφ after 3 hours (**Figure 1C**). Using microscopy or flow cytometry we noticed a decreasing fluorescence signal intensity originating from AC, suggesting that Mφ digest the apoptotic material over time. We then analyzed the mRNA expression of classical LXR target genes, i.e., ABCA1 and ABCG1 as well as PPARδ/γ targets pyruvate dehydrogenase kinase 4 (PDK4) and carnitine palmitoyltransferase 1A (CPT1A) (**Figure 1D**). Expression of LXR and PPAR mRNA targets peaked at 6-9 hours post-efferocytosis and returned to baseline after 24 hours. Therefore, we choose 6 hours (3 hours exposure to AC, followed by 3 hours after their removal) for subsequent gene expression analyses. Q-PCR analyses of genes specifically expressed in Jurkat cells (CD3E, CD3D, LCK) revealed that the amount of Jurkat mRNA did not exceed 1% of total mRNA in the samples. Furthermore, Jurkat cells showed negligible ABCA1 and PDK4 mRNA expression as compared to macrophages. Therefore, we excluded significant contribution of RNA from apoptotic cells in Mφ. Data so far confirm rapid activation of PPAR and LXR target genes in efferocytotic human Mφ.

### RNA-Seq Analysis of Efferocytotic Human Mφ

**Figure 2:**
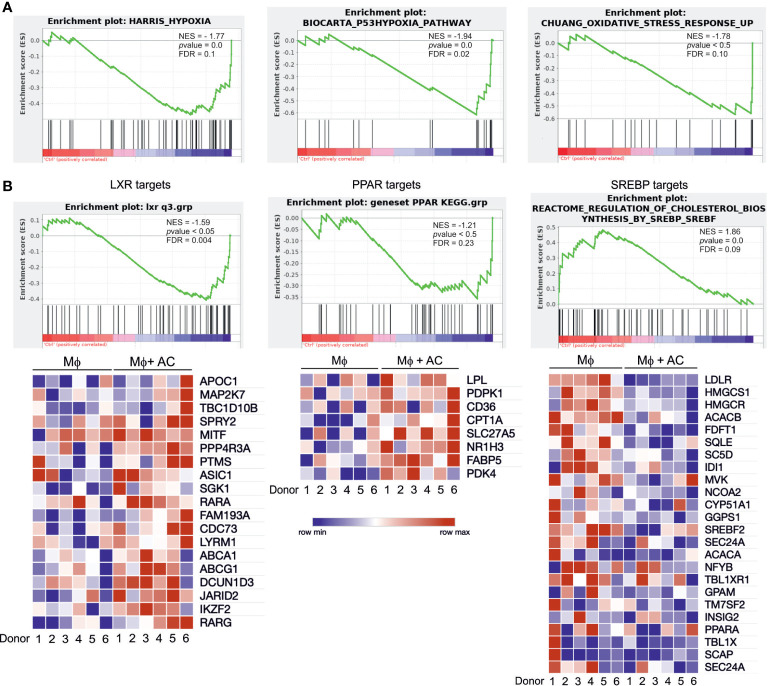
RNA-seq analysis of efferocytotic human Mφ. **(A)** GSEA plots of positively and negatively regulated metabolic pathways in human Mφ 6 hours post-efferocytosis of apoptotic Jurkat cells. Green curves indicate enrichment scores. The normalized enrichment score (NES), p value, and false discovery rate (FDR, cut-off value<0.25) are indicated within each graph. **(B)** GSEA plots and heatmaps of positively regulated LXR and PPAR target genes as well as negatively regulated SREBP-2 targets. Each square of the heatmaps represents one biological replicate. Blue and red color indicate down vs. upregulated genes, respectively.

To explore global transcriptional responses of efferocytotic human Mφ we performed RNA-seq analysis using 6 biological replicates. Again, Mφ were exposed to AC for 3 hours, followed by AC removal and subsequent incubations for 3 hours. To group differentially expressed, functional subsets of genes, we performed gene set enrichment analysis (GSEA). Efferocytotic Mφ exhibited transcriptional changes related to several metabolic and stress-signaling pathways. We noticed upregulation of oxidative stress responses and hypoxia pathways, as previously reported by us and others (15–17) (**Figure 2A**). Moreover, in addition to upregulating genes referring to PPAR and LXR pathways, GSEA data revealed strong downregulation of SREBP-2 targets associated with cholesterol biosynthesis and sterol metabolism (**Figure 2B**). Conclusively, changes in the activity of PPARs, LXRs and SREBP-2 point to the importance of lipid-related changes in efferocytotic Mφ.

### Activation of LXR and PPARδ Targets in Efferocytotic Mφ

**Figure 3:**
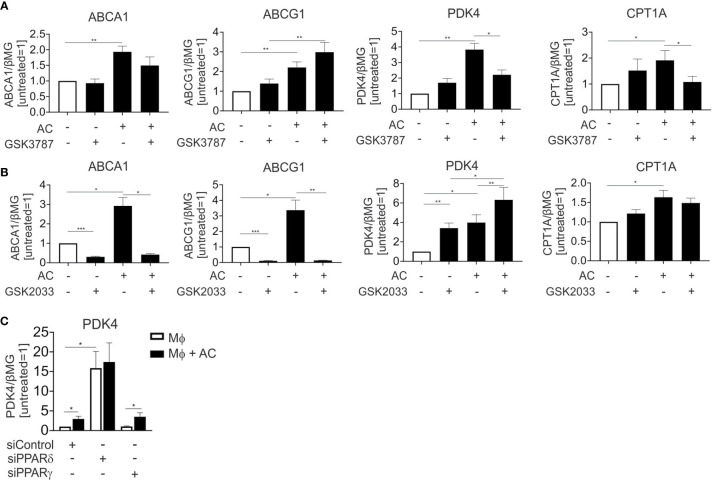
LXR and PPARδ antagonists block target gene induction in efferocytotic Mφ. **(A, B)** mRNA expression of ABCA1, ABCG1, PDK4 and CPT1A in Mφ treated with 1 µM GSK3787 **(A)** or 5 µM GSK2033 and AC **(B)** for 6 hours. **(C)** PDK4 mRNA expression in Mφ transfected with PPARδ and PPARγ siRNA for 96 hours and subsequently exposed to AC for 6 hours. *P ≤ 0.05; **P ≤ 0.01; ***P ≤ 0.001.

LXR and PPAR transcription factor families consist of two (LXRα and LXRβ), respectively three (PPARα, PPARγ, and PPARδ) members. In human Mφ LXRα is predominantly expressed (18) and linked to efferocytotic responses, while both PPARγ and PPARδ are equally expressed and are known to regulate efferocytosis-induced gene expression in various experimental systems. To understand the relative contribution of PPARδ, PPARγ and LXRs to individual target gene expression during efferocytosis, we inhibited PPARδ and LXRs during the uptake of apoptotic Jurkat cells with the PPARδ antagonist GSK3787 (19) or the LXR antagonist GSK2033 (20) followed by mRNA expression analysis of ABCA1, ABCG1, PDK4, and CPT1A. GSK3787 prevented the induction of PDK4 and CPT1A in efferocytotic Mφ, while expression of ABCA1 and ABCG1 was not affected (**Figure 3A**). In contrast, GSK2033 suppressed ABCA1 and ABCG1 expression but not that of PDK4 or CPT1A (**Figure 3B**). Strikingly, GSK2033 increased PDK4 expression in the absence of AC, suggesting that this inhibitor might activate PPARδ. This appears rational as GSK2033 had been described as a ligand for multiple nuclear receptors (21) and considering that GSK3787 attenuated effects of GSK2033 (**Supplementary Figure 1A**). PDK4 is still induced by AC in the presence of GSK2033 (**Figure 3B**). PDK4 mRNA is also induced by the synthetic PPARγ ligand rosiglitazone (**Supplementary Figure 1B**), but this induction is insensitive to GSK3787, confirming the specificity of the latter one as a PPARδ antagonist. To further explore the contribution of PPARγ and PPARδ to the induction of PDK4, we silenced these transcription factors in efferocytotic Mφ. Consistent with the repressive function of PPARδ in the absence of a ligand (22), knocking down PPARδ substantially upregulated PDK4 expression (**Figure 3C**). AC failed to induce PDK4 RNA in PPARδ-silenced cells, confirming a PPARδ-dependence of PDK4 induction in efferocytotic Mφ. In contrast, silencing PPARγ preserved a PDK4 mRNA increase in response to AC, indicating that PPARγ does not substantially contribute to PDK4 induction upon efferocytosis. Unfortunately, we could not use the pharmacological PPARγ antagonists GW9662 or T0070907, since both of them elicited strong PDK4 induction, probably through PPARδ activation (23) (data not shown). Collectively, our data suggest that AC increase PDK4 and CPT1A mRNA expression through PPARδ, whereas ABCA1 and ABCG1 are induced through LXRs.

### Lysosomal Phospholipase PLA2G15 Generates PPARδ Ligands in Efferocytotic Mφ

**Figure 4:**
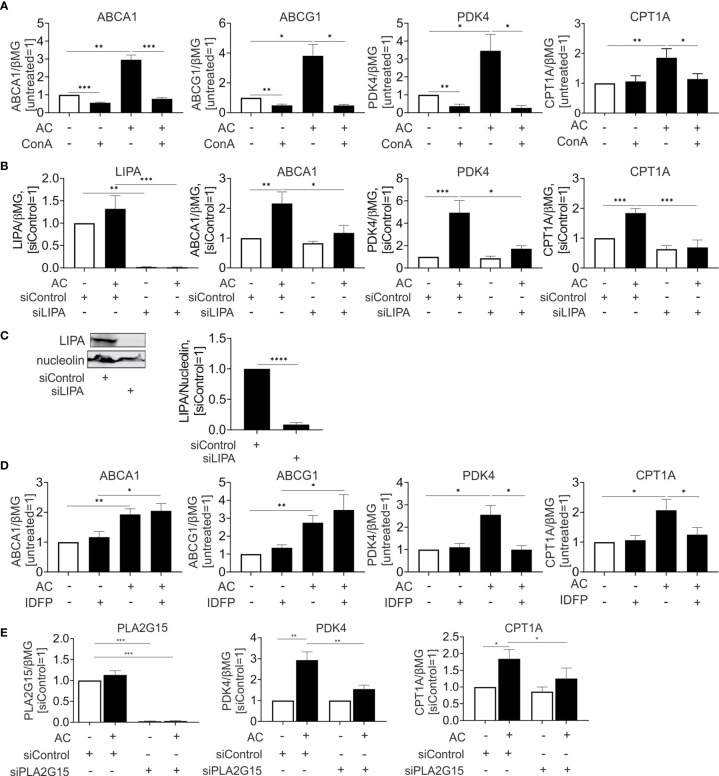
Lysosomal phospholipase PLA2G15 generates PPARδ ligands. **(A, D)** mRNA expression of indicated targets in Mφ treated with 100 nM concanamycin A (ConA) **(A)** or 10 µM IDFP **(D)** in combination with AC for 6 hours. **(B, E)** mRNA expression of indicated targets in Mφ transfected with LIPA **(B)** or PLA2G15 **(D)** siRNA for 96 hours and subsequently treated with AC for 6 hours. **(C)** Western analysis of LIPA expression transfected with LIPA siRNA for 96 hours. *P ≤ 0.05; **P ≤ 0.01; ***P ≤ 0.001; ****P ≤ 0.0001.

Currently, activation of PPARs and LXRs upon efferocytosis is only incompletely understood. We hypothesized that fatty acids and sterols, liberated upon lysosomal digestion of ingested material, might cause their transcriptional activation. To address this possibility, we inhibited lysosomal acidification using the lysosomal v-ATPase inhibitor concanamycin A. Blocking lysosomal acidification interfered not only with induction of LXR targets ABCA1 and ABCG1, but also the PPARδ targets PDK4 and CPT1A (**Figure 4A**). Conclusively, lysosomal processing of apoptotic cells is a pre-requisite for LXR and PPARδ activation.

Recently, Viaud and colleagues (14) suggested lysosomal acid lipase (LIPA) as an essential component to generate LXR ligands in efferocytotic THP-1 cells. To explore the relevance of this pathway for primary human Mφ we silenced LIPA with over 90% efficiency both at the mRNA and protein level (**Figures 4B, C**). In agreement with published observations (14), LIPA silencing prevented induction of the LXR target ABCA1 mRNA by apoptotic cells. Strikingly, a LIPA knockdown also interfered with PPARδ target gene expression in efferocytotic Mφ (**Figure 4B**). Thus, LIPA is critical for processing ingested material to generate both LXR and PPARδ ligands. Also, the hydrolysis of ingested phospholipids by lysosomal phospholipase A_2_ (PLA2G15) may generate PPAR activating ligands. To inhibit PLA2G15, we used isopropyl dodecylfluorophosphonate (IDFP), a covalent modifier of the catalytic serine-165 residue (24). As seen in **Figure 4D**, IDFP lowered PDK4 and CPT1A expression in efferocytotic MΦ, with no regulation of ABCA1 or ABCG1. To verify the role of PLA2G15, we used a knockdown strategy and analyzed mRNA expression of PPARδ targets in efferocytotic Mφ (**Figure 4E**). Silencing PLA2G15 in Mφ indeed attenuated PDK4 and CPT1A expression.

### Antagonizing LXR Attenuates the ABCA1-Facilitated Cholesterol Efflux From Efferocytotic Mφ

Previous studies suggested that LXRs and PPARs play important roles in controlling the expression of efferocytotic receptors, cholesterol efflux, and suppressing inflammatory responses in efferocytotic Mφ (8, 25). However, most of these studies employed Mφ with a constitutive knockout of these transcription factors, with an altered expression of efferocytotic receptors or cholesterol transporters. We took advantage of acute pharmacological inhibition of LXRs and PPARδ during efferocytosis to examine how LXR and PPARδ activation in efferocytotic macrophages affects cholesterol efflux, mitochondrial metabolism, and inflammatory response.

**Figure 5:**
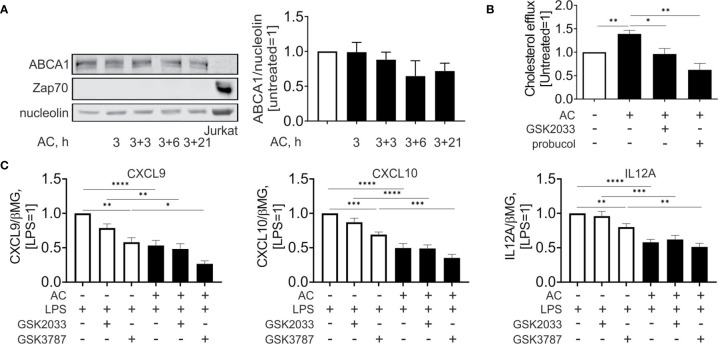
Effect of LXR and PPARδ agonists on cholesterol efflux and inflammatory responses upon efferocytosis. **(A)** Western analysis of ABCA1 and Zap70 expression in Mφ following efferocytosis of AC an in Jurkat cells. **(B)** Cholesterol efflux from efferocytotic Mφ to ApoAI-containing medium in the presence or absence of GSK2033 or probucol. **(C)** mRNA expression of CXCL9, CXCL10, and IL12A in Mφ co-treated with AC, LPS, GSK2033, and GSK3787 for 3 hours. *P ≤ 0.05; **P ≤ 0.01; ***P ≤ 0.001; ****P ≤ 0.0001.

Surprisingly, and contrary to mRNA expression data, we noticed that protein expression of ABCA1 (**Figure 5A**) was not significantly altered during efferocytosis. Analysis of Jurkat-specific protein Zap70 showed no contamination of the samples with proteins from engulfed cells. Nevertheless, when we analyzed cholesterol efflux to apoAI in the supernatant of Mφ 24 hours post-efferocytosis (**Figure 5B**), we observed an increased cholesterol accumulation in apoAI-containing medium. This increase is sensitive to the co-treatment with GSK2033 or the ABCA1 inhibitor probucol. Apparently, pharmacological LXR inhibition acutely suppresses ABCA1-mediated cholesterol efflux from efferocytes.

PDK4 and CPT1A support mitochondrial fatty acid oxidation through inhibition of the mitochondrial pyruvate dehydrogenase complex (PDH) and maintaining fatty acid transport into mitochondria, respectively. While we were unable to find commercial antibodies specifically detecting PDK4 in human Mφ, we did not observe changes in PDH phosphorylation upon efferocytosis (**Supplementary Figure 2A**). Similarly, CPT1A protein remained unaltered in efferocytotic Mφ (**Supplementary Figure 2A**). Finally, analysis of mitochondrial oxygen consumption and extracellular acidification rates using Seahorse extracellular flux analysis showed no alterations in efferocytotic Mφ, suggesting that efferocytosis doesn’t significantly affect mitochondrial metabolism in human Mφ (**Supplementary Figure 2B**).

To assess the importance of LXR and PPARδ in modulating the inflammatory profile of efferocytotic Mφ, we acutely inhibited these transcription factors using GSK2033 and GSK3787, respectively, and simultaneously treated Mφ with lipopolysaccharide (LPS) and apoptotic cells (**Figure 5C**). LPS stimulated the mRNA expression of inflammatory chemokines, i.e., chemokine (C-X-C motif) ligand 9 and 10 (CXCL9 and CXCL10) as well as the pro-inflammatory interleukin 12A (IL12A). In efferocytotic Mφ expression of CXCL9/10 and IL12A mRNA was attenuated. Whereas PPARδ inhibition by GSK3787 attenuated the expression of these targets in LPS-stimulated Mφ, apoptotic cells still exhibited anti-inflammatory effects in the presence of GSK2033 or GSK3787. We did not observe significant alterations of anti-inflammatory IL10 in efferocytotic Mφ under these conditions (**Supplementary Figure 2C**). In summary, these observations indicate that apoptotic cells attenuate LPS-induced inflammatory gene expression probably independently of LXR or PPARδ agonsim.

## Discussion

In this study, we characterized changes in gene expression using a human efferocytosis model of primary Mφ phagocytosing apoptotic Jurkat cells. RNA sequencing of efferocytotic Mφ revealed transcriptional changes, referring to various metabolic and stress-signaling pathways that previously had been noticed by us and others (15–17). Thus, efferocytotic Mφ upregulated glucose transporters SLC2A1 and SLC2A3 as well as other glycolytic genes, likely a consequence of hypoxia-induced factor 1α activation (15). Furthermore, we noticed upregulation of heme oxygenase-1, confirming our previous observations (26), as well as induction of a p53 transcriptional target CDKN1A. These stress responses may attenuate inflammatory activation of Mφ upon apoptotic cell engulfment (26). Our data also show that LXRs, PPARs and SREBPs are the main transcriptional regulators of lipid metabolism in efferocytotic human Mφ. A strong downregulation of SREBP-2 targets was previously observed in efferocytotic LR73 hamster phagocytes (17), and in human efferocytotic Mφ (27). In human Mφ, accumulation of sterol biosynthetic intermediates upon efferocytosis likely contributes to LXR activation and SREBP-2 suppression (27). In addition to sterol homeostasis targeted by LXRs and SREBP-2, fatty acid metabolism undergoes transcriptional regulation in efferocytotic Mφ through PPARs. Interestingly, pharmacological and genetic interventions of PPARδ suggested that this transcription factor is dominant in activating lipid metabolic genes upon efferocytosis, while PPARγ targets do not appear to contribute. Obviously, PPARδ is the main PPAR target during efferocytosis in the human setting.

Mechanisms how PPARs and LXRs are activated upon efferocytosis are not completely understood. Our data suggest that lysosomal processing of apoptotic cells is a pre-requisite for LXR and PPAR activation. Whereas the activity of LIPA was essential for generating LXR ligands in efferocytotic THP-1 cells (14), in our model the knockdown of LIPA suppressed induction of PPARδ as well as LXR target genes. These findings are surprising, considering the LIPA shows some specificity towards digestion of neutral lipids, such as cholesterol esters and triglycerides, which are not the predominant lipid species in efferocytosed Jurkat cells. Since LIPA inhibition did not impair lysosomal acidification and proteolytic activity (14), we did not expect a general defect of lysosomal function in LIPA-silenced Mφ. The exact mechanism how LIPA generates PPARδ/LXR ligands thus remains enigmatic and necessitates further research.

Besides LIPA, we showed that pharmacological inhibition or a knockdown of PLA2G15 abolished induction of PPARδ target genes. Conclusively, PPARδ activation in efferocytotic Mφ demanded the processing of engulfed phospholipids with the release of free fatty acids by lysosomal PLA2G15. These observations point to a pivotal role of lysosomal digestion in PPARδ activation during AC clearance. Nonetheless, additional studies are necessary to explore the nature of lysosome-derived ligands necessary for PPAR and LXR activation.

LXR regulates cholesterol efflux to apoAI-containing medium (28, 29). Our observations suggest that pharmacological LXR inhibition in Mφ acutely suppresses ABCA1-mediated cholesterol efflux. Although we could not observe upregulation of total ABCA1 protein, LXR might enhance the ABCA1 transporting capacity in efferocytotic Mφ, perhaps by specifically increasing the expression of the efflux-promoting pool of ABCA1 protein.

The PDH complex is responsible for adjusting the metabolic flexibility in mammals (30). PDH activity is suppressed through phosphorylation, catalyzed by four highly specific PDK isozymes (30). The most widely expressed are PDK2 and PDK4 in heart, liver and kidney of humans and rodents (30). Inactivation of PDH by upregulating PDK4 is known to shift glucose catabolism to fatty acid utilization (30, 31). Here, we show the PPARδ target PDK4 to be one of the strongest upregulated gene upon efferocytosis. Nevertheless, we failed to observe metabolic changes as described before (30, 31), and moreover, could not detect an altered PDH phosphorylation. Additionally, PDH phosphorylation in Mφ was insensitive to PDK4 silencing or upregulation by the PPARδ agonist GW501516, but was blocked by the PDK inhibitor dichloroacetate (**Supplementary Figure 3**). Conclusively, PDK4 is not a major regulator of PDH phosphorylation in human macrophages.

Besides regulating AC uptake, LXR and PPARs are also implicated to attenuate inflammatory responses during efferocytosis (25, 32). In LXR-deficient Mφ the production of anti-inflammatory cytokines, i.e. TGFβ or IL-10 upon AC engulfment as compared to the wild-type Mφ is impaired (25). A LXR deficiency also failed to suppress LPS-induced IL-1β and IL-12 expression by AC. Similarly, PPARδ-deficient efferocytotic Mφ displayed reduced IL-10 and elevated TNFα and IL-12 secretion upon LPS-stimulation (8). In our experimental setting AC still attenuated LPS-induced inflammatory gene expression, even when LXR and PPARδ were inhibited. This may reflect the lack of IL-10 and TGFβ induction by AC under our conditions as well as differences between human and the mouse system. Our observations indicate that AC are able to attenuate LPS-induced inflammatory gene expression probably independently of LXR or PPARδ agonism.

In summary, our findings present novel mechanistic insights on LXR and PPARδ activation in human efferocytotic Mφ. At the same time, our work highlights notable differences in the impact of PPARδ and LXR inhibition on the metabolism and anti-inflammatory phenotype of efferocytes as compared to rodent knockout models that deserve more attention and clarification in the future research.

## Data Availability Statement

The datasets presented in this study can be found in online repositories. The names of the repository/repositories and accession can be found below: https://www.ncbi.nlm.nih.gov/geo/query/acc.cgi?acc=GSE169160.

## Author Contributions

AM conceived and performed the experiments, analyzed the data, and drafted the manuscript. MD, AW, and RS contributed to data analysis and interpretation. DN and BB participated in study design, data analysis, and writing of the final manuscript draft. All authors contributed to the article and approved the submitted version.

## Funding

This study was supported by the grants from Deutsche Forschungsgemeinschaft (SFB 1039, Teilprojekt A05, B04, B06 and BR999/25-1).

## Conflict of Interest

The authors declare that the research was conducted in the absence of any commercial or financial relationships that could be construed as a potential conflict of interest.
