# Supplementary material for: Lysosome-Dependent LXR and PPARδ Activation Upon Efferocytosis in Human Macrophages
Source: Front Immunol. 2021 May 7;12:637778. doi: 10.3389/fimmu.2021.637778 (PMC8137840; doi:10.3389/fimmu.2021.637778)
Supplement: Supplementary file 1 [file DataSheet_1.pdf]

Supplementary Figures

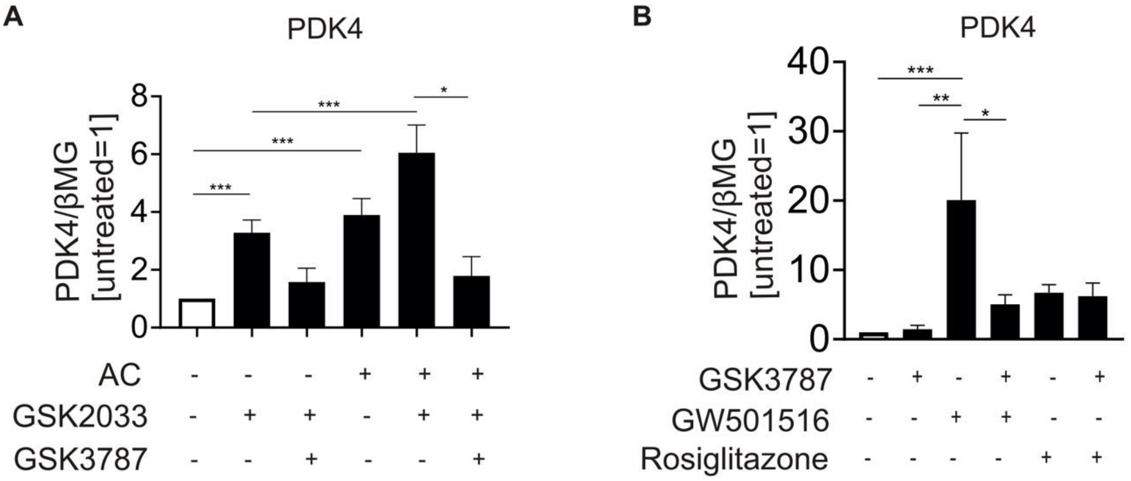

Supplementary Figure 1.

(A) mRNA expression of PDK4 in Mϕ treated with 1 µM GSK3787, 5 µM GSK2033 or both in combination with apoptotic Jurkat cells (AC) for 6 hours. (B) mRNA expression of PDK4 in Mϕ treated with 1 µM GSK3787, 100nM GW501516 and 1 µM Rosiglitazone. *P≤0.05; **P≤0.01; ***P≤0.001.

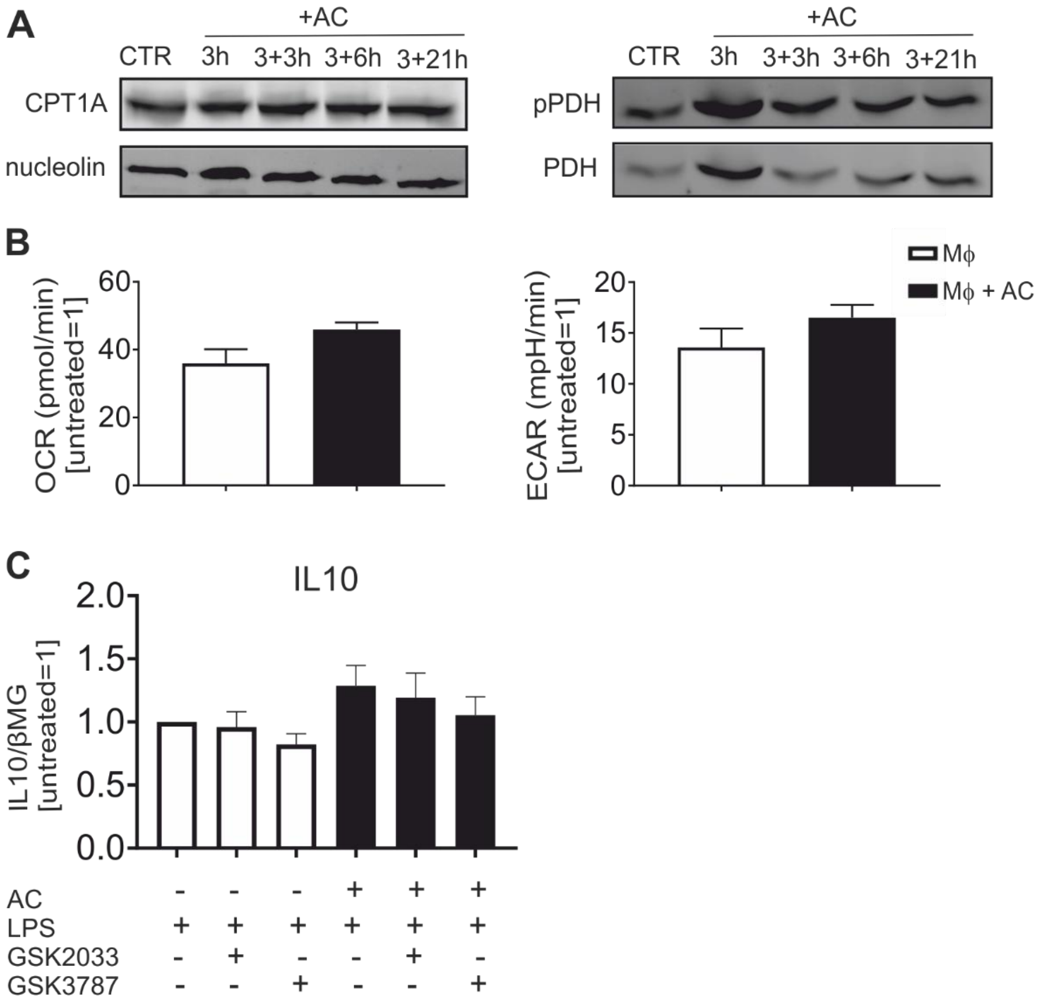

Supplementary Figure 2.

(A) Protein analysis of CPT1A, PDH and phosphorylated PDH (pPDH) in Mϕ at indicated time points post-efferocytosis. (B) Basal oxygen consumption rate (OCR) and extracellular acidification rate (ECAR) of Mϕ co-incubated with AC for 6 hours. (C) mRNA expression of IL10 in Mϕ co-treated with AC, LPS, GSK2033 and GSK3787 for 3 hours.

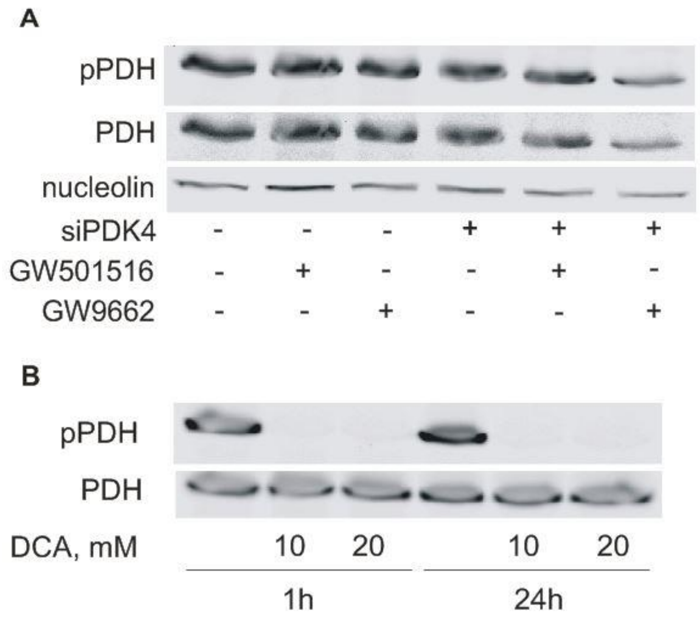

Supplementary Figure 3.
(A) Protein analysis of PDH and phosphorylated PDH (pPDH) in Mϕ following transfection with siPDK4 for 96 hours and subsequent treatment with 100 nM GW501516 and 5 µM GW9662 for 24 hours. (B) Analysis of PDH and pPDH in Mϕ treated with 10 or 20 mM PDK inhibitor dichloroacetate (DCA) for 1 or 24 hours.
